# Genotype-ocular biometry correlation analysis of eight primary angle closure glaucoma susceptibility loci in a cohort from Northern China

**DOI:** 10.1371/journal.pone.0206935

**Published:** 2018-11-06

**Authors:** Wenjuan Zhuang, Shaolin Wang, Juan Hao, Manyun Xu, Hao Chi, Shunyu Piao, Jianqing Ma, Xiaolong Zhang, Shaoping Ha

**Affiliations:** 1 People’s Hospital of Ningxia Hui Autonomous Region, Ningxia Eye Hospital (First Affiliated Hospital of Northwest University For Nationalities), Yinchuan, China; 2 Clinical Medical College of Ningxia Medical University, Yinchuan, China; 3 Department of Ophthalmology, Taiyuan Central Hospital, Taiyuan, China; 4 Shandong Academy of Medical Sciences, Jinan University, Jinan, China; 5 Department of Ophthalmology, Wuzhong People's Hospital, Wuzhong, China; Bascom Palmer Eye Institute, UNITED STATES

## Abstract

**Purpose:**

Recent genome-wide association studies (GWAS) have verified eight genetic loci that were significantly associated with primary angle–closure glaucoma (PACG). The present study investigated whether these variants are associated with the ocular biometric parameters of anterior chamber depth (ACD) and axial length (AL) in a northern Chinese population, as well as whether there were differences in the association of genetic markers in our cohort based on ethnicity.

**Methods:**

A case-control association study of 500 patients and 720 controls was undertaken. All individuals were genotyped for eight single nucleotide polymorphisms (SNPs) (rs11024102 in *PLEKHA7*, rs3753841 in *COL11A1*, rs1015213 located between *PCMTD1* and *ST18*, rs3816415 in *EPDR1*, rs1258267 in *CHAT*, rs736893 in *GLIS3*, rs7494379 in *FERMT2*, and rs3739821 mapping between *DPM2* and *FAM102A*) using an improved multiplex ligation detection reaction (iMLDR) technique. Allelic and genotypic frequency differences were evaluated using a logistic regression model. Generalized estimation equation (GEE) analysis was conducted for association testing between genotypes and ocular biometric parameters. False discovery rate (FDR) correction for multiple comparisons was employed, and the statistical power was calculated via power and sample size calculation.

**Results:**

Four of the eight SNPs, rs3753841, rs1258267, rs736893 and rs7494379, were associated with PACG (p = 0.007, 0.0016, 0.0045, 0.045, respectively), and only rs3753841, rs1258267 and rs736893 surpassed the FDR correction. For subgroup analysis, only rs1258267 could withstand multiple testing correction in the Han nationality (p = 0.00571). In the GEE tests, rs3753841, rs1258267 and rs736893 were found to be nominally associated with ACD (p = 0.023, 0.016, 0.01, respectively). However, these associations could not survive FDR correction.

**Conclusions:**

The SNP rs3753841 in *COL11A1*, rs1258267 in *CHAT* and rs736893 in *GLIS3* are associated with PACG in northern Chinese people, and the association of genetic markers manifests a tendency of ethnic diversity. Larger population-based studies are warranted to reveal additional PACG loci and ethnic aspects of PACG.

## Introduction

Primary angle-closure glaucoma (PACG) is a common subtype of glaucoma, which is characterized by appositional approximation or contact between the iris and trabecular meshwork, resulting in obstruction of aqueous outflow and intraocular pressure elevation [1]. In 2013, the number of people with PACG worldwide was estimated to be 11.7 million, which will increase to 21 million in 2020, and the number of people estimated to be bilaterally blind from PACG in 2020 will increase to nearly 5.3 million [2]. More importantly, epidemiological studies have revealed that most PACG cases are in Asia [3], especially in China [4]. Furthermore, PACG has long been thought to have a significant genetic basis, although the exact disease-causing mutations have not been identified [5,6], and geographic and racial variation in the prevalence of PACG was validated [7]. To date, genome-wide association studies (GWAS) have verified eight genetic loci significantly associated with PACG: *EPDR1*, *CHAT*, *GLIS3*, *FERMT2*, *DPM2-FAM102A*, *PLEKHA7*, *COL11A1*, and *PCMTD1-ST1*8 [8,9]. In addition, eye configuration is perhaps the other most prominent risk factor for PACG. In a recent GWAS of anterior chamber depth (ACD) and PACG in an Asian cohort, variants in the ATP-binding cassette, subfamily C, member 5 (*ABCC5*) encoding gene were found to be related with ACD and increased risk of PACG development [10].

Collectively, data from these studies suggest that geographic and racial variations, multiple genes and anatomical risk factors help to determine the potential risk for developing PACG. However, the exact mechanisms by which they influence PACG risk have not been thoroughly elucidated to date. Importantly, whether these genetic loci are associated with the anatomical risk factors, ocular biometric parameters of ACD and axial length (AL), in a specific population is unknown. Consequently, the purpose of this study was to investigate whether the eight PACG susceptibility loci identified by the GWAS contribute to development of PACG by modifying PACG endophenotypes such as AL and ACD in a northern China population. In particular, we are interested in whether the association of implicated genetic markers would be diverse in different ethnic groups.

## Materials and methods

### Subjects

The present case-control study included 500 PACG patients and 720 ethnically matched unaffected controls recruited from Ningxia Eye Hospital in the northern regions of China. This study was approved by the local hospital’s ethics committee acting in accordance with the tenets of the Declaration of Helsinki and written informed consent was obtained from all study participants. Comprehensive ophthalmic examinations were performed for each participant, including best-corrected visual acuity, intraocular pressure (IOP) measurement, slit lamp biomicroscopy, fundus photography, gonioscopy, visual field and ultrasound biomicroscopy. ACD and AL were measured in both cases and controls by noncontact partial coherence interferometry using the Zeiss IOL Master Optical Biometer (IOLMaster, Carl Zeiss Meditech Ltd.). Five measurements of AL and ACD (defined as corneal epithelium to anterior crystalline lens surface) were obtained and the mean value was taken for further statistical analysis. The diagnosis of PACG in patients was in accord with the International Society of Geographical and Epidemiological Ophthalmology (ISGEO) [11], based on the presence of glaucomatous optic neuropathy with a cup-to-disc ratio equal to or more than 0.7, peripheral visual loss, IOP more than 21 mmHg, and the presence of at least 180° of closed angle in which the trabecular meshwork is not visible by gonioscopy. Patients with a previous history of cataractous operations were excluded in our study. The control group were ethnically matched individuals who were required to have none of the above characteristics (having an intraocular pressure of less than 21 mmHg, with open angles in all quadrants, healthy optic nerves, and normal visual fields), no known family history of glaucoma, no previous glaucomatous or cataractous operations and no other ophthalmic diseases besides mild senile cataracts. Participants with secondary angle closure glaucoma caused by uveitis, trauma, or neovascularization were excluded.

### DNA extraction

Peripheral venous blood samples were collected from all participants using EDTA-containing tubes. Then, whole DNA was extracted from the blood samples utilizing the Simgen DNA Blood Mini Kit (Simgen, Hangzhou, China) according to the manufacturer’s protocol. The extracted DNA was eluted in TE buffer (10 mM Tris-HCl, 0.5 mM EDTA, pH 9.0) and stored at -80°C until use after the A260/A280 optical density was measured with a Nanodrop2000 (Thermo Fisher Scientific Inc., Wilmington, DE, USA).

### SNP selection and genotyping

Eight single nucleotide polymorphisms (SNPs) were selected through GWA studies since they were the most firmly replicated loci: rs11024102 (*PLEKHA7*), rs3753841 (*COL11A1*), rs1015213 (*PCMTD1-ST18*), rs3816415 (*EPDR1*), rs1258267 (*CHAT*), rs736893 (*GLIS3*), rs7494379 (*FERMT2*) and rs3739821 (*DPM2- FAM102A*). They were then genotyped by Genesky Biotechnologies Inc. (Shanghai, China) using an improved multiplex ligation detection reaction (iMLDR) technique.

### Statistical analysis

All analyses were performed using SPSS software (version 17.5: *SPSS* Science, Chicago, IL). Differences in gender and ethnicity between cases and controls were assessed by the χ2 test and differences in age between the two groups were assessed by t test. Hardy-Weinberg equilibrium (HWE) of each SNP was analyzed using the χ2 test. The genetic association analyses were conducted using PLINK (version 1.07; http://pngu.mgh.harvard.edu/~purcell/plink/, in the public domain). Differences in the allelic and genotypic frequencies of a given SNP were evaluated and adjusted by age and gender using a logistic regression model. Meanwhile, adjusted odds ratios (ORs) and the corresponding 95% confidence intervals (CIs) for associations were also presented. Meta-analysis of the two different ethnic groups was conducted by PLINK using the age- and gender-adjusted logistic regression results. Generalized estimation equation (GEE) analysis with an unstructured working correlation matrix model for a trend-per-copy effect on the minor allele (coding 0 for the wild-type genotype, 1 for heterozygous carriers of the minor allele, and 2 for individuals homozygous for the minor allele) was performed using SPSS for primary association testing between genotypes and ocular biometric parameters in the case group, in which the genotypes were treated as covariates, ACD and AL were the control variables of each other, and age and gender were adjusted. The false discovery rate (FDR) method [12] was used for multiple testing correction in both GEE analysis and genetic association studies. The statistical power was calculated by the Power and Sample Size Calculation (PS; version 3.1.232).

## Results

In total, 500 PACG patients (147 males and 353 females; 93 Hui and 407 Han) and 720 control subjects (332 males and 388 females; 129 Hui and 591 Han) were enrolled in this study. There was no significant difference in ethnicity between the cases and controls. However, the control subjects were significantly older (mean age 71.82 ± 7.2 years vs. 63.77 ± 9.576 years; p = 0.000) and included fewer women (53.9% vs. 70.6%; p = 0.000) than the cases group (Table 1).

**Table 1:** Demographic characteristics of PACG cases and controls.

	Cases	Controls	*P*
Number	500	720	
Age, y (Mean ± SD)	63.77 ± 9.576	71.82 ± 7.2	0.000#
Sex, n (%)			0.000*
Male	147 (29.4)	332 (46.1)	
Female	353 (70.6)	388 (53.9)	
Nationality, n (%)			0.761*
Han	407(81.4)	591 (82.1)	
Hui	93 (18.6)	129 (17.9)	

#The *p*-value was tested by t-test.

*The *p*-value was assessed by χ2 test.

Overall, the genotyping call rates for the eight SNPs in both case and control groups were more than 99% and no SNPs deviated from HWE (p>0.05) (Table 2). Among the eight SNPs, significant genetic associations with PACG were identified for rs3753841 in *COL11A1* (p = 0.007), rs1258267 in *CHAT* (p = 0.0016), rs736893 in *GLIS3* (p = 0.0045) and rs7494379 in *FERMT2* (p = 0.045) after correction for age and gender using logistic regression, and rs3753841, rs1258267 and rs736893 survived after FDR for multiple testing correction (Table 2).

**Table 2:** Association of target SNPs with PACG after adjustment for age and gender.

GENE	SNP	CHR	BP	Minor allele	Genotype (AA/AB/BB)*		MAF		HWE-*p*		OR (95%CI)	*P*
					Case	Control	Case	Control	Case	Control		
*PCMTD1–ST18*	rs1015213	8	52887541	T	476/24/0	695/25/0	0.024	0.0174	1	1	1.234 (0.65 ~ 2.34)	0.52
*PLEKHA7*	rs11024102	11	17008605	C	156/236/108	257/331/132	0.452	0.413	0.321	0.1668	1.148 (0.96 ~1.37)	0.131
*COL11A1*	rs3753841	1	103379918	G	215/227/58	342/318/60	0.343	0.3043	0.921	0.2904	1.31 (1.075 ~ 1.6)	0.007
*EPDR1*	rs3816415	7	37988311	A	389/101/10	568/139/13	0.121	0.1142	0.289	0.1969	1.068(0.8125~1.405)	0.6354
*CHAT*	rs1258267	10	50895770	G	301/176/23	374/292/54	0.222	0.2772	0.7955	0.8526	0.7085(0.5719~0.8776)	0.0016
*GLIS3*	rs736893	9	4217028	A	284/186/30	374/310/63	0.246	0.3036	1	0.659	0.7415(0.6031~0.9115)	0.00452
*FERMT2*	rs7494379	14	53411391	T	250/196/54	312/325/83	0.304	0.3412	0.1124	0.9341	0.8217(0.6779~0.9959)	0.04528
*DPM2–FAM102A*	rs3739821	9	130702477	G	239/208/53	366/293/61	0.314	0.2876	0.4673	0.8558	1.104(0.9072~1.344)	0.3229

*A represents the wild-type allele, B represents the minor allele; CHR, chromosome; BP, base pair position; MAF, minor allele frequency; HWE-*p*, the *p*-value of Hardy-Weinberg equilibrium

*p*-value and OR, CI were calculated with a logistic regression model by adjusting for age and gender

We also performed a subanalysis within the Hui patients versus Hui controls and Han patients versus Han controls, since the recruited participants included two ethnic groups. Rs3753841 in *COL11A1* (p = 0.0344) and rs736893 in *GLIS3* (p = 0.0144) were nominally associated with PACG in the Hui cohort, and by the same token, rs1258267 in *CHAT* (p = 0.0057), rs736893 in *GLIS3* (p = 0.0279) and rs7494379 in *FERMT2* (p = 0.0329) were nominally associated with PACG in the Han cohort after correction for age and gender using logistic regression. However, after FDR correction, only rs1258267 in *CHAT* remained significantly associated with PACG in the Han cohort. A meta-analysis of the two different ethnicities was then performed, in which rs3753841 in *COL11A1* (I^2^ = 0.9%, fixed-effects meta-analysis *p*-value = 0.0069), rs1258267 in *CHAT* (I^2^ = 0, fixed-effects meta-analysis *p*-value = 0.0015) and rs736893 in *GLIS3* (I^2^ = 38.28%, fixed-effects meta-analysis *p*-value = 0.0024) still showed significant association with PACG (Table 3).

**Table 3:** Associations of target SNPs between cases and controls in different ethnicities and meta-analysis results.

GENE	SNP	Genotype (AA/AB/BB)*				OR (95%CI)		*P*		P-meta*	I^2^	*P-het*
		Case (HUI)	Case (HAN)	Control (HUI)	Control (HAN)	HUI	HAN	HUI	HAN			
*PCMTD1–ST18*	rs1015213	85/8/0	391/16/0	125/4/0	570/21/0	2.673(0.7114~10)	0.9394(0.4454~1.981)	0.1442	0.8697	0.5645	45.3	0.1764
*PLEKHA7*	rs11024102	28/46/19	128/190/89	41/57/31	216/273/102	0.9852(0.6641~1.462)	1.21(0.9887~1.481)	0.941	0.06439	0.107	0	0.3635
*COL11A1*	rs3753841	37/41/15	178/186/43	60/59/10	281/260/50	1.603(1.035~2.481)	1.247(0.9989~1.557)	0.0344	0.0512	0.006992	0.9	0.3151
*EPDR1*	rs3816415	74/18/1	315/83/9	108/17/4	461/121/9	1.072(0.5815~1.977)	1.08(0.7942~1.468)	0.8235	0.6242	0.5969	0	0.9872
*CHAT*	rs1258267	63/25/5	238/151/18	72/48/9	302/245/44	0.6806(0.4178~1.109)	0.7146(0.5631~0.9069)	0.1223	0.00571	0.001571	0	0.8604
*GLIS3*	rs736893	57/34/2	227/152/28	58/57/14	289/253/49	0.5454(0.3357~0.886)	0.7729(0.6144~0.9724)	0.01437	0.02787	0.002401	38.28	0.2031
*FERMT2*	*rs7494379*	*47/31/15*	*203/165/39*	59/61/9	*252/265/74*	1.034(0.6763~1.581)	0.7906(0.6371~0.9811)	0.8772	0.03287	0.06688	18.05	0.2693
*DPM2–FAM102A*	rs3739821	38/43/12	201/165/41	49/60/20	317/233/41	0.9224(0.6089~1.397)	1.157(0.9243~1.449)	0.7028	0.2031	0.3484	0	0.3467

MAF, minor allele frequency; OR, odds ratio; CI, confidence interval; I^2^: measures heterogeneity, p-het: *p*-value for heterogeneity; *p*-value and OR, CI were calculated with logistic regression model by adjusting for age and gender.

*P-meta, *p*-value obtained by meta-analysis, if the I^2^ value was ≥50%, take the value of random-effects; otherwise, a fixed-effects model was adopted.

In the next analysis, the association between the eight SNP genotypes and AL and ACD ocular biometric parameters in the case group was evaluated using GEE tests. We found rs3753841 in *COL11A1*, rs1258267 in *CHAT* and rs736893 in *GLIS3* were nominally associated with the ACD (p = 0.023, 0.016 and 0.01, respectively). Among the three SNPs, the variant alleles may have the effect of shallowing the ACD (β = -0.046) for rs3753841 in *COL11A1* and deepening the ACD for rs1258267 in *CHAT* and rs736893 in *GLIS3* (β = 0.054 and 0.058, respectively) (Table 4). Nevertheless, none of the SNPs surpassed FDR correction for multiple testing in GEE analysis.

**Table 4:** Association between the eight PACG susceptibility loci and AL and ACD.

GENE	SNP	Minor allele	AL (22.92 ± 0.891;20.01~25.51)*			ACD (2.74±0.474;0.25~4.51)*		
			*β*	SE	*P*	*β*	SE	*P*
*PCMTD1–ST18*	rs1015213	T	-0.020	0.0878	0.821	-0.043	0.0588	0.464
*PLEKHA7*	rs11024102	C	-0.022	0.030	0.470	-0.012	0.0184	0.510
*COL11A1*	rs3753841	G	-0.013	0.0317	0.692	-0.046	0.0201	0.023
*EPDR1*	rs3816415	A	-0.034	0.0447	0.453	-0.035	0.0256	0.173
*CHAT*	rs1258267	G	0.043	0.0352	0.225	0.054	0.0223	0.016
*GLIS3*	rs736893	A	0.057	0.0335	0.091	0.058	0.0223	0.010
*FERMT2*	rs7494379	T	0.007	0.0313	0.830	0.033	0.0191	0.088
*DPM2–FAM102A*	rs3739821	G	-0.018	0.0336	0.603	-0.009	0.0205	0.678

* Numbers in parentheses indicate the mean ± SD and the range of measured values for AL or ACD

*β*, per-allele effect in ACD/AL; SE, standard error for ascertainment of *β*; *P*, *p*-value for association adjusted for age and gender.

The power varies between the eight SNPs due to differences of their minor allele frequency (MAF) and allelic OR value. Therefore, our sample size provides 62.5% to 93.6% statistical power to detect a significant association at an α level of 0.05.

## Discussion

A case-control study to examine the association between eight established PACG susceptibility loci and anatomical risk factors in a cohort from northern China was conducted. We found rs1258267 in *CHAT* and rs736893 in *GLIS3* were consistently significantly associated with PACG, which so far has not been replicated in other studies except in a correlation between five novel genetic loci and primary angle closure suspect (PACS) investigated by Monisha et al. [13]. Rs3753841 in *COL11A1* was another locus which was observed to be associated with PACG in our cohort. The contribution of the associated SNP was also confirmed in patients from Australia and Nepal [14]. Rs3753841 is a missense mutation which is considered likely to tag another functional variant responsible for PACG, rather than being a causative allele [14]. Rs1258267 in *CHAT* and rs736893 in *GLIS3* were reported as new genetic loci through an expanded GWAS and are involved as candidate pathways in the development of PACG [9]. *CHAT* is on chromosome 10 and encodes choline acetyltransferase, which is supposed to influence the risk for PACG by influencing acetylcholine metabolism, while *GLIS3* is a member of the GLI-similar subfamily of Krüppel-like zinc-finger proteins, which is implicated in currently unknown metabolic pathways mediated through zinc-finger activation that could contribute to PACG pathogenesis [9,15]. These variants are likely to provide new insights into molecular mechanisms leading to PACG and further work is needed to determine the functional effects of these variants on PACG susceptibility.

Similarly, another association was identified between genotype and ACD and AL ocular biometry using the GEE method with binocular measurement data [16,17]. Rs3753841 in *COL11A1* (p = 0.023; OR 1.31) correlated with shallower ACD with a 0.046 mm per-allele effect, while rs1258267 in *CHAT* (p = 0.016; OR 0.709) and rs736893 in *GLIS3* (p = 0.01; OR 0.742) correlated with deeper ACD with a 0.054 mm and 0.058 mm per-allele effect, respectively. For these three loci, the direction of the effect predisposing towards angle closure development was consistent in the ORs’ values. However, none of the three SNPs surpassed FDR correction for multiple testing. Even so, we speculated that rs3753841, rs1258267 and rs736893 could predispose to PACG by affecting the ACD ocular biometric parameter, since the *p*-values were very close to 0.05 after FDR correction (*p*-values were 0.06, 0.06 and 0.08, respectively). The FDR correction is commonly applied in association studies to evaluate the significance of multiple testing of SNPs, but it may lead to loss of significant findings because it is known to be conservative for positively correlated *p*-values [12]. For this reason, a considerably larger case-control study of the significance of tag SNPs and appropriate multiple-correction criteria were applied, which would be a more definitive way to determine a real association in a separate population. Interestingly, none of the novel PACG loci showed significant association with AL in our study, which provides some hints that genetic polymorphisms in these genes could predispose to PACG by affecting ACD rather than AL through our limited data. This result likely supports the view of most specialists that ACD is the primary risk factor for angle closure glaucoma [18]. Previous studies have demonstrated that ocular biometric parameters such as shallow ACD and short AL are strong risk factors for PACG [19–21]. Wang and colleagues revealed that approximately one in ten Chinese individuals with narrow angles developed PACG [22]. Nevertheless, it is difficult to predict which eyes with shallow anterior chambers will develop glaucomatous features using anatomic or imaging features alone. Thus, we hypothesized that shallow ACD combined with the positive genetic variants would enhance an individual’s risk of developing PACG. Exploring the genetic markers associated with PACG as well as anatomical risk factors in large independent cohorts may therefore be vital for better understanding the mechanisms involved in the disease process.

Furthermore, subgroup analyses were conducted to determine the association of these eight genes with PACG in different ethnic groups, since the participants recruited in our study included Hui and Han nationalities. Different from the abovementioned results, rs3753841 in *COL11A1* and rs736893 in *GLIS3* were nominally associated with PACG in the Hui cohort while rs1258267 in *CHAT*, rs736893 in *GLIS3* and rs7494379 in *FERMT2* were nominally associated with PACG in the Han cohort after correction for age and gender. Only rs736893 was a mutual nominally associated genetic locus and the odds ratios in the two different ethnic groups were in the right direction. This likely reflected ethnic differences in disease pathogenesis and implied the association of markers is diverse in different ethnic groups despite the insufficient sample size of Hui people. After FDR correction, only rs1258267 in *CHAT* remained significantly associated with PACG in the Han cohort. Thus, ethnic heterogeneity should be considered in further replication studies to determine the association of genetic loci with PACG including varying nationality, especially in China, as China is a multiethnic country. Certainly, such results should be replicated in an independent cohort for further validation and interpreted cautiously due to the limited data pertaining to PACG in the two ethnic groups, which might provide insufficient statistical power to reveal the association of the ethnic-difference with PACG. Given that our study involves two ethnic groups, which may have an impact on our combined analysis, a meta-analysis of the two different ethnic groups was performed. The *p*-values were almost the same as in the initial overall analysis, providing strong evidence to verify our initial overall analysis.

There are several other limitations to this study. First, only the AL and ACD were included for analysis of the association between SNPs and ocular biometric parameters. Other important imaging-based anatomical risk factors for angle closure which can provide us with more information to understand its pathogenesis, such as anterior chamber width, anterior chamber volume, iris thickness and lens vault, were not obtained. Meanwhile, the obtained data on ocular biometric parameters were not population-based, which would introduce some bias in sample selection. Second, for some loci, due to the relatively low minor allele frequency detected in our cohort, the statistical power is less than 70%, such as in rs1015213, as our sample size was not large enough to identify its possible associations. Third, since clarification of genetic risk factors for different stages of PACG can be helpful in early diagnosis and prognosis in clinical practice, a larger sample size, including primary angle closure (PAC) patients, will be needed to detect the association in early stages.

In summary, our study validates the association between the findings of a large GWAS implicating *COL11A1*, *CHAT* and *GLIS3* genes in PACG in a northern Chinese population. Furthermore, by looking at a variety of genetic marker associations in different ethnic groups, our observations manifested a tendency of ethnic-difference. These findings support prior reports of the association of *COL11A1*, *CHAT* and *GLIS3* with PACG and the importance of the contribution of genetic heterogeneity to the development of PACG. Additionally, the lack of associations between the susceptibility loci with ACD and AL suggests that more biochemical features involved in PACG risk should be assessed in disease pathogenesis. Further research investigating the association between PACG susceptibility loci and other anatomical and physiological risk factors of angle closure in additional independent cohorts involving PAC patients with an adequate sample size is warranted.
